# Supplementary material for: Ultralow mode-volume photonic crystal nanobeam cavities for high-efficiency coupling to individual carbon nanotube emitters
Source: Nat Commun. 2014 Nov 25;5:5580. doi: 10.1038/ncomms6580 (PMC4263150; doi:10.1038/ncomms6580)
Supplement: Supplementary Information — Supplementary Figure 1 and Supplementary Note 1. [file ncomms6580-s1.pdf]

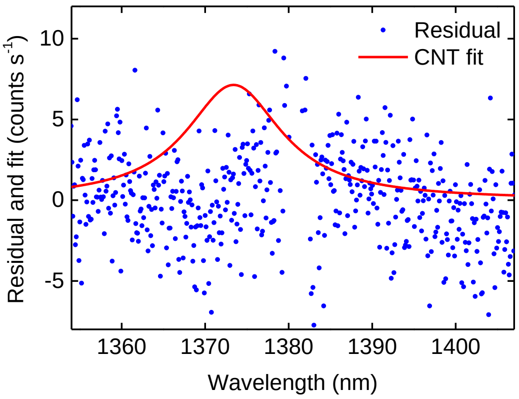

Supplementary Figure 1 | Fitting error analysis. From the data shown in Figs. 4a and 4b, residual of the fit is obtained by subtracting the bi-Lorentzian fit (blue dots). The carbon nanotube (CNT) peak obtained from the fit (red curve) is plotted on the same scale for comparison. The CNT peak height is approximately 7 counts s^{-1}, while the residual in a 10-nm window centered around the CNT peak has a standard deviation of 2.97 counts s^{-1} and a standard error of mean of 0.302 counts s^{-1}. The CNT peak value is more than 23 times larger than the standard error of mean, indicating a statistically significant result.

Supplementary Note 1

Using the fact that photoluminescence intensities are proportional to the emission rate and the collection efficiency under steady-state conditions, $I_{\mathrm{cav}} = A\,\eta_{\mathrm{cav}}\,\gamma_{\mathrm{cav}}$ and $I_{\mathrm{CNT}} = A\,\eta_{\mathrm{CNT}}\,\gamma_{\mathrm{CNT}}$, where A is a proportionality constant. The spontaneous emission coupling factor is then given by

$$\beta = \frac{\gamma_{\mathrm{cav}}}{\gamma_{\mathrm{CNT}} + \gamma_{\mathrm{cav}}} = \frac{I_{\mathrm{cav}}/(A\,\eta_{\mathrm{cav}})}{I_{\mathrm{CNT}}/(A\,\eta_{\mathrm{CNT}}) + I_{\mathrm{cav}}/(A\,\eta_{\mathrm{cav}})} = \frac{I_{\mathrm{cav}}}{(\eta_{\mathrm{cav}}/\eta_{\mathrm{CNT}})I_{\mathrm{CNT}} + I_{\mathrm{cav}}}. \quad (1)$$
